# Association of flavored electronic nicotine delivery system (ENDS) use with self-reported chronic obstructive pulmonary disease (COPD): Results from the Population Assessment of Tobacco and Health (PATH) study, Wave 4

**DOI:** 10.18332/tid/127238

**Published:** 2020-10-01

**Authors:** Hangchuan Shi, Zahíra Quiñones Tavárez, Zidian Xie, Liane M. Schneller, Daniel P. Croft, Maciej L. Goniewicz, Scott McIntosh, Richard J. O’Connor, Deborah J. Ossip, Irfan Rahman, Dongmei Li

**Affiliations:** 1 Department of Clinical and Translational Research, University of Rochester Medical Center, Rochester, United States; 2 Department of Public Health Sciences, University of Rochester Medical Center, Rochester, United States; 3 Department of Pulmonary and Critical Care Medicine, University of Rochester Medical Center, Rochester, United States; 4 Department of Health Behavior, Roswell Park Comprehensive Cancer Center, Buffalo, United States; 5 Department of Environmental Medicine, University of Rochester Medical Center, Rochester, United States

**Keywords:** PATH, ENDS, flavors, COPD

## Abstract

**INTRODUCTION:**

Flavors other than tobacco flavor have been identified as a major reason for electronic nicotine delivery system (ENDS) initiation in youth and are thought to contribute to the continued use of ENDS in users of all ages. Our previous research showed a significant association between overall ENDS use and COPD. This study aims to identify the association of ENDS flavor categories with self-reported COPD.

**METHODS:**

The data analysis included 4909 adults from Population Assessment of Tobacco and Health (PATH) Wave 4 data who were ever established ENDS users and responded to an item about diagnosis of COPD. Weighted multivariable logistic regression models were used to examine the association between different ENDS flavors and self-reported COPD considering complex sampling design.

**RESULTS:**

Among 4909 ever established ENDS users, 418 adults (weighted percentage 9.8%) had self-reported COPD. Self-reported COPD prevalence differed between different ENDS flavor categories, with the highest (weighted percentage 19.9%) occurring among tobacco flavor users. Compared to non-tobacco flavor categories, tobacco flavor category showed significantly higher association with self-reported COPD (AOR=2.05; 95% CI: 1.20–3.53), after adjusting for potential confounding variables. No significant associations with self-reported COPD were found for other examined ENDS flavor categories including menthol/mint, fruit, candy/ desserts/other-sweets, and other flavors, compared to their corresponding non-users.

**CONCLUSIONS:**

Tobacco flavored ENDS use was significantly associated with self-reported COPD. Future studies are needed to confirm the biological and epidemiological association of flavored ENDS use with COPD.

## INTRODUCTION

Electronic nicotine delivery systems (ENDS) are handheld devices that heat a liquid containing mixture of solvents and flavoring chemicals, with or without nicotine, to produce an aerosol. The prevalence of ENDS use among young adults (18–24 years) increased 46.2% from 2017 to 2018 (5.2% vs 7.6%; respectively), similar to the 48.5% and 77.8% increments among middle and high school students in the US^1^. Powerful marketing strategies by ENDS companies have been associated with ENDS being the most common tobacco product in the youth population^2^. Moreover, flavors other than tobacco have been identified as a major reason for ENDS initiation in youth and are thought to contribute to the continued use in users of all ages^3,4^. In addition, flavors are associated with a lower perception of ENDS harmful effects^5^. The short- and long-term health impacts of ENDS use that are currently being studied include a variety of outcomes similar to those described for combustible cigarettes, e.g. addiction^6,7^, cardiovascular disease^8-10^ and respiratory diseases^11^. The recent multi-state outbreak of ‘e-cigarette or vaping product use-associated lung injury (EVALI)’ involved more than 2600 cases (as of January 2020) and raised national concern about the acute harmful effects of ENDS^12^. According to the US Centers for Disease Control and Prevention (CDC), tetrahydrocannabinol (THC, the psychoactive ingredient in cannabis or marijuana) delivered with Vitamin E acetate in ENDS has been linked to many of the EVALI documented cases^13^.

There is an incipient but growing body of evidence showing the detrimental effects of ENDS flavors on respiratory health. Though there is lack of evidence of the long-term health effects of ENDS use, exposure to ENDS has been associated with pulmonary damage as shown by *in vitro* animal and human studies^14^. Exposure to flavored ENDS aerosol has been shown to trigger oxidative distress and inflammation among lung epithelial cells, which in turn are related to asthma and chronic obstructive pulmonary disease (COPD)^15,16^. Our previous research using national survey data identified a significant association of overall ENDS use with self-reported COPD^17^ and COPD-related respiratory symptoms^18^. Recent published studies showed that chemicals used in several ENDS flavor categories (mint, vanilla, and other) elicited an increased mitochondrial oxidative stress response in bronchial cells and disrupted the epithelial barrier by increasing inflammatory intermediates^19,20^. Additionally, a pilot human study showed that the use of fruit and sweet flavors resulted in an augmented systemic immune response to clinical respiratory symptoms^21^. However, less is known about the effects of different ENDS flavors in the development and progression of COPD. This study aims to identify the association of ENDS flavor categories with self-reported COPD by analyzing the Population Assessment of Tobacco and Health (PATH) Study Wave 4 data.

## METHODS

### Study population

The PATH study began in September 2013 (Wave 1). It is a nationally representative population-based study of people aged ≥12 years^22^. With the collection of data from sequential annual Waves, the PATH study is designed to provide both cross-sectional and longitudinal assessment of patterns of tobacco product use and associated health effects. Weighting procedures were used to adjust for selection variations and differential non-response rates. Further details regarding the PATH study design can be found elsewhere^22,23^. For this study, we used the PATH Wave 4 adult dataset collected from 1 December 2016 to 3 January 2018 from 33822 adults (aged ≥18 years).

In the current analyses, we calculated the crosssectional estimates from 4909 adult participants who ever established use of ENDS. The group of ever established ENDS users has two components: former established ENDS users and current established ENDS users. Using the PATH Wave 4 derived variables, we defined former established ENDS users (2921 adults) as those who have ever used any ENDS fairly regularly, have not used them within the past 12 months or are currently not using them. Respondents who have ever used any ENDS and currently use them every day or some days were defined as current established ENDS users (1988 adults). The same definition of ENDS user in the PATH study has been used in publications elsewhere^18^.

### Data availability

The datasets are de-identified open-source data that are publicly available from the National Addiction & HIV Data Archive Program (NAHDAP) website (https://www.icpsr.umich.edu/icpsrweb/NAHDAP/ studies/36498/datadocumentation).

### Assessment of dependent variable

A flowchart (Supplementary file, Figure S1) shows the process of how we classified COPD cases. Additionally, according to the definition of COPD used by World Health Organization (WHO)^24^, people with self-reported emphysema or chronic bronchitis were also considered to have COPD in this study. We identified those with self-reported COPD at Wave 4 from the following question: ‘Has a doctor, nurse or other health professional told you that you have COPD?’ (Q1); and ‘You told us you had COPD. Are you troubled by shortness of breath when hurrying on level ground or walking up a slight hill?’ (Q2). Participants were asked the second question (Q2) if they were: 1) a continuing adult respondent who ever reported, at Waves 1 to 3, being diagnosed with COPD; 2) a continuing adult respondent who did not have COPD as of their last interview, but have been told they had COPD in the past 12 months; or 3) they were newly enrolled respondents at Wave 4 who have ever been told they had COPD. Because there is an option ‘I have never had COPD’ to the second question (Q2), we used this question to identify the respondents who ever falsely self-reported COPD. Thus, respondents who answered, ‘I have never had COPD’ to this question, combined with participants who responded ‘No’ to the first question (Q1), were classified as not having COPD. People with emphysema or chronic bronchitis were identified as individuals who answered ‘Yes’ to the question: ‘Has a doctor, nurse or other health professional told you that you have 1) emphysema or 2) chronic bronchitis?’.

### Assessment of independent variables

Participants who were ever ENDS users were asked: ‘What flavor is/was your regular brand or the brand you last used? Choose all that apply’. The response options provided nine flavor categories including: tobacco-flavored, menthol/mint, clove/spice, fruit, chocolate, an alcohol drink, a non-alcohol drink, candy/desserts/other-sweets, or some other flavor. Tobacco-flavored, menthol/mint, fruit, and candy/desserts/other-sweets are of interest because they were reported as the four most commonly used flavors based on our previous study^4^. All other flavor categories were combined as a single flavor category defined as ‘other flavors’. Thus, five binary flavor use variables were created: tobacco-flavored (yes/ no), menthol/mint (yes/no), fruit (yes/no), candy/ desserts/sweets (yes/no), and other flavors (yes/ no). In consideration of multiple flavor users, we examined pairwise correlations among the five flavor use categories and showed the matrix of Pearson correlation coefficients (r) (Supplementary file, Table S1). Due to the small correlations (|r| < 0.34), we considered flavor categories as independent variables.

Traditional cigarette smoking status (e.g. nonsmoker, former smoker, and current smoker) was categorized using Wave 4 derived variables. Current established cigarette smokers were those who reported to have smoked at least 100 cigarettes in their lifetime, and currently smoke every day or some days. Former smokers were defined as those who have smoked at least 100 cigarettes in their lifetime, and have not smoked them within the past 12 months or currently smoke not at all. Non-smokers were those who smoked fewer than 100 cigarettes in their lifetime. The category was based on the standard definition used by the Centers for Disease Control and Prevention (CDC)^25^.

All adult respondents who have ever used ENDS were asked: ‘Have you ever used marijuana, marijuana concentrates, marijuana waxes, THC, or hash oils in an electronic product?’. Those who responded ‘yes’ were subsequently asked: ‘When you have used an electronic product, how often were you using it to smoke marijuana, marijuana concentrates, marijuana waxes, THC, or hash oils?’. According to the responses to these two questions, we categorized ‘marijuana use in ENDS’ as five levels: never, rarely, sometimes, most of the time, and every time.

In addition to tobacco smoking and marijuana use, other covariates were chosen based on previous studies on the association between ENDS and COPD^26,27^ and CDC reports on COPD^28^. All variables were categorized into variables with two or more levels. The examined covariates include age, sex, race/ ethnicity, history of asthma, and household income.

### Statistical analysis

We used weighted frequency distributions and the Rao-Scott modified likelihood ratio test to examine the association between dependent and independent variables. Multivariable weighted logistic regression models were then used to investigate the adjusted association of ENDS flavor use with self-reported COPD. The balanced repeated replication (BRR) method was used to form replicate weights with Fay’s adjustment of 0.3. Weighted percentages, adjusted odds ratios (AORs) and their 95% confidence intervals (CIs) were reported. Analyses were conducted using SAS v9.4 (SAS Institute Inc., Cary, NC), with a significance level for two-sided tests set at 5%.

Five separate multivariable weighted logistic regression models were conducted. Model 1, stratified by ENDS use status plus smoking status, and included all five flavor use variables as predictors. Model 2, stratified by ENDS use status, included all five flavor use variables as predictors, and controlled for smoking status. Model 3, controlled for all variables in Model 2 plus age, sex, race/ethnicity, marijuana use in ENDS, history of asthma, and household income. Finally, two additional stratified models based on Model 3 were created to examine the potential modification effects of asthma (Model 4) and sex (Model 5).

## RESULTS

Of the 4909 ever established ENDS users, the overall prevalence of self-reported COPD was 9.8% (95% CI: 4.6–19.7%). Among these ENDS users, 20.6% (95% CI: 10.8–35.6%) reported tobacco flavor use, 21.6% (95% CI: 13.3–33.2%) reported menthol/mint flavor use, 45.0% (95% CI: 29.8–61.2%) reported fruit flavor use, 30.7% (95% CI: 18.0–47.3%) reported candy/deserts/sweets flavor use, and 13.6% (95% CI: 6.1–27.7%) reported other flavors use. Of ever established ENDS users, 37.4% (95% CI: 20.6–57.9%) reported using more than two of the above five flavor categories. The prevalence of self-reported current smoking was 58.7% (95% CI: 46.8–69.7%) and selfreported marijuana use in ENDS was 26.1% (95% CI: 16.8–38.1%).

In the Supplementary file, Table S2 shows the characteristics of ever established ENDS users across different flavors. The prevalence of self-reported COPD was the highest (19.9%) among tobacco flavor users, and lowest among fruit flavor users (5.3%) and candy/desserts/sweets flavor users (5.1%). Current smokers were most likely to use tobacco flavor (67.1%) than any other flavor. Non-smokers were most likely to use fruit flavor (20.4%), but least likely to use tobacco flavor (4.7%). Former smokers did not show any significant ENDS flavor use preference. In addition, tobacco flavor users were more likely to be older adults (≥45 years) and non-Hispanic White. Menthol/mint flavor users were more likely to be non-Hispanic Black and Hispanic, and to have lower household income. Fruit and candy/desserts/sweets flavor users were more likely to be younger adults, and to use marijuana in ENDS.

In the Supplementary file, Table S3 shows the weighted distribution of the study population according to COPD outcome stratified by smoking status. Among ever established ENDS users, nonsmokers, former smokers and current smokers who ever reported COPD were more likely to use tobacco flavor (29.8%, 43.8% and 47.3%; respectively). However, former smokers and current smokers without self-reported COPD were less likely to use tobacco flavor (18.8% and 21.8%; respectively), but more likely to use fruit flavor (46.1% and 43.1%; respectively). The weighted distribution of the study population according to COPD outcome stratified by ENDS use status is shown in Table S4 (Supplementary file). Results from the weighted test showed that selfreported COPD was associated with tobacco flavor, fruit flavor and candy/desserts/sweets flavor along with other listed covariates.

Tables 1 and 2 show the AORs of self-reported COPD prevalence estimated in regression Models 1, 2 and 3. In Model 1, among current smokers, ever established ENDS users who regularly used tobacco flavors had 1.43 times higher odds of self-reported COPD (AOR=2.43; 95% CI: 1.56–3.86), compared with those who were not tobacco flavor users (Table 1). Among this same group of current smokers who had ever established ENDS use, use of fruit flavor was associated with somewhat lower odds of self-reported COPD (AOR=0.63; 95% CI: 0.43–0.93), and use of other flavors was associated with somewhat higher odds of self-reported COPD (AOR=1.89; 95% CI: 1.04–3.44). Although ever established ENDS users who never smoked had higher odds of self-reported COPD when they were tobacco flavor users versus non-tobacco flavor users, the total number of selfreported COPD cases in this group (Supplementary file, Table S3) was too small (n=23) to provide a reasonable confidence interval. Similar patterns with those among ever established ENDS users were seen among the current established ENDS users subset (Supplementary file, Table S5). No significant associations of any flavor use with self-reported COPD were observed among former smokers who were ever established ENDS use.

**Table 1:** Adjusted ORs* and 95% CI of self-reported COPD associated with ENDS flavors among ever established ENDS users stratified by smoking status, Model 1

*Flavors*	*Ever established ENDS users*		
	*Non-smoker*	*Former smoker*	*Current smoker*
Tobacco	**12.36 (2.10–73.16)**	2.24 (0.74–6.76)	**2.43 (1.56–3.86)**
Menthol or mint	0.85 (0.13–5.66)	1.09 (0.34–3.50)	1.05 (0.68–1.61)
Fruit	2.39 (0.39–14.59)	0.50 (0.14–1.80)	**0.63 (0.43–0.93)**
Candy, desserts or sweets	1.90 (0.21–16.92)	0.59 (0.24–1.49)	0.48 (0.33–0.72)
Other	0.19 (0.01–5.68)	0.96 (0.39–2.37)	**1.89 (1.04–3.44)**

* Odds of self-reported COPD among users of a specific flavor versus odds of self-reported COPD among non-users of this specific flavor as reference. The adjusted ORs were controlled for the effects of all flavors other than the interested specific flavor.

In Model 2, ever established ENDS users who regularly used tobacco flavors were associated with higher odds of self-reported COPD (AOR=2.64; 95% CI: 1.75–3.98), compared with those who were not tobacco flavor users. However, the relationship was attenuated with further adjustment for sex, age, race/ethnicity, income, history of asthma, and use of marijuana in ENDS in Model 3 (Table 2). Stratified analysis showed that in Model 2 among the current established ENDS users stratum, regular tobacco flavor use was associated with more than 3-fold greater odds of self-reported COPD (AOR=3.23; 95% CI: 2.02–5.17) compared with non-tobacco-flavored ENDS users. The relationship remained significant after adjusting for additional confounders in Model 3 (AOR=2.05; 95% CI: 1.20–3.53). In Model 2, current established ENDS users who regularly used other flavors were associated with somewhat higher odds of self-reported COPD versus those who did not use other flavors (AOR=2.10; 95% CI: 1.08–4.11). However, this significance was lost after further adjustment in Model 3. We did not observe significant associations between fruit or any other flavor use and self-reported COPD. Stratified analyses by history of asthma and sex are shown in Tables S6 and S7 (Supplementary file). No significant modification effects were observed.

**Table 2:** Adjusted ORs* and 95% CI of self-reported COPD associated with ENDS flavors stratified by ENDS use status

*ENDS use status*	*Tobacco*	*Menthol or mint*	*Fruit*	*Candy, desserts or sweets*	*Other*
**Model 2**					
Ever established	**2.64 (1.75–3.98)**	1.09 (0.72–1.64)	0.66 (0.43–1.00)	0.58 (0.41–0.82)	1.53 (0.95–2.47)
Former established	1.90 (0.95–3.80)	0.84 (0.37–1.89)	0.67 (0.29–1.51)	0.49 (0.24–1.02)	0.80 (0.36–1.77)
Current established	**3.23 (2.02–5.17)**	1.30 (0.78–2.17)	0.66 (0.40–1.07)	0.63 (0.38–1.04)	**2.10 (1.08–4.11)**
**Model 3**					
Ever established	1.50 (0.93–2.42)	1.0 (0.56–1.81)	0.81 (0.49–1.35)	0.77 (0.53–1.14)	1.10 (0.71–1.72)
Former established	0.99 (0.40–2.51)	0.67 (0.20–2.29)	0.81 (0.24–2.75)	0.54 (0.15–2.00)	0.66 (0.23–1.90)
Current established	**2.05 (1.20–3.53)**	1.30 (0.67–2.52)	0.88 (0.50–1.55)	0.98 (0.58–1.65)	1.56 (0.79–3.08)

* Odds of self-reported COPD among users of a specific flavor versus odds of self-reported COPD among non-users of this specific flavor as reference. The adjusted ORs in Model 2 were controlled for the effects of all other flavors, and smoking status. The adjusted ORs in Model 3 were controlled for the effects of all other flavors, smoking status, history of asthma, sex, age, race/ethnicity, income level, and marijuana use in ENDS.

## DISCUSSION

The observed overall self-reported COPD prevalence among ever established ENDS users (9.8%) in this study was greater than the general prevalence reported in the US (3.9–7.5%)^29,30^. Two previous studies, using the PATH Wave 1 dataset^26^ and the BRFSS dataset^27^, respectively, reported COPD prevalence similar to the current study, and demonstrated the association of ENDS use with higher odds of self-reported COPD. However, we observed an even higher self-reported COPD prevalence among ever established ENDS users who regularly used tobacco flavor (19.9%).

The major finding from this current cross-sectional study is that there is an association between tobacco flavor use in ENDS and self-reported COPD. Among current smokers, individuals who were ever established ENDS users had significantly increased odds of self-reported COPD if they regularly used tobacco flavor in ENDS. Among individuals who currently use ENDS every day or some days, the regular use of tobacco flavor was associated with significantly higher odds of self-reported COPD, regardless of whether they were non-smokers, former smokers or current smokers.

COPD is characterized by lung inflammation^31^. Previous studies have shown that exposure of lungs to ENDS can cause an inflammatory response by triggering the secretion of cytokines, inducing oxidative stress, and altering innate immune elements^32-34^. However, increasing biological evidence suggests the possibility that tobacco flavor liquid may serve as an independent inducer of lung inflammation in addition to the baseline risk of ENDS. Our previous study has shown that tobacco flavored e-liquid was an independent source of oxidants or reactive oxygen species (OX/ROS) in addition to common ENDS liquid components such as vegetable glycerin (VG) and propylene glycol (PG)^35^. Allen et al.^36^ tested 51 flavored e-liquids and found that some tobacco flavored e-liquids contained diacetyl and 2,3-pentanedione, which are well known to be associated with bronchiolitis obliterans (‘popcorn lung’). A recent *in vitro* study tested lethal doses of different e-liquid flavors and showed that tobacco flavor liquid was much more toxic than VG or PG^37^. These studies indicated that the lung inflammation and injury caused by tobacco flavor liquid may independently contribute to the development of COPD. Though *in vitro* studies have observed an increased inflammatory response from menthol flavored e-liquids compared to tobacco flavored e-liquids, we did not find any significant association of menthol/mint flavor with self-reported COPD in this study. Thus, more studies, both biological and epidemiological, are needed in the future to confirm the association.

### Limitations

One major limitation in this study is the lack of ability to determine causality. Our cross-sectional study design does not allow for causal inferences, and although the expected direction of the explanation is that tobacco flavored ENDS users are more likely to have self-reported COPD, the reverse direction of the explanation may also contribute to the finding of the higher odds of self-reported COPD. Specifically, it is possible that current smokers with self-reported COPD were more likely to use a tobacco flavor because they may have only partially switched to ENDS in the hope of reducing the progression of COPD common to traditional cigarettes. These smokers may choose tobacco-flavored ENDS because it reminds them of their former combustible habit. Indeed, Tables S2 and S3 (Supplementary file) showed some predictable evidence: 1) older people were more likely to use tobacco flavor; 2) older people were more likely to report COPD; and 3) current smokers without selfreported COPD were less likely to use tobacco flavor in ENDS. Those findings supported the statement of the reverse direction of the explanation. However, results from the models in this study suggest the expected direction of the explanation. In Model 1, the significant higher odds of self-reported COPD associated with the use of tobacco flavored ENDS were seen among both current smokers and never smokers (Table 1). In Models 2 and 3, the significant associations were stable after adjusting for age and smoking status (Table 2). These results indicate that tobacco flavored ENDS users are more likely to have self-reported COPD independent of age and smoking history. Additionally, based on PATH Wave 3 data with a large overlap of the population in this study, our previous study has shown that there was no association between type of flavor category and cigarette smoking quit attempts^4^. This evidence suggests that tobacco flavor may not be the factor affecting a participant’s decision to switch to ENDS.

Another noticeable limitation in this study is that the diagnosis of COPD is based on self-report by respondent in the PATH survey. It can be a source of recall bias and may result in misclassification. Even in clinical settings, the lack of diagnosis by spirometry and clinical assessment may result in improper diagnosis of COPD^38^. For example, smokers with productive cough may self-report as COPD^39^, leading to overestimation of COPD cases in this study. To minimize the potential misclassification, we used multiple survey questions about COPD (e.g. self-reported diagnosis, exercise tolerance reduction etc.) to define COPD cases, rather than relying on a single Yes/No question (Supplementary file, Figure S1). However, the misclassification is an intrinsic limitation of studies based on survey data.

Other limitations in this study are noted. First, although PATH has a large sample size, the cell size is relatively small when we focused on self-reported COPD cases in a specific stratum, which limited our statistical power. The small cell size also limited our ability to: 1) further control for some potential confounders (e.g. other tobacco products); and 2) further examine the association with COPD based on disease severity (e.g. stage of disease, exacerbation by infection etc.) and comorbidity (e.g. lung cancer, cardiovascular diseases etc.). Second, we cannot identify the chemical ingredients of each flavor. Since chemical additives may differ largely between brands, we cannot estimate the variation within each flavor category.

Interpreted with a number of limitations in mind, our results are still informative and relevant to the current and future knowledge base. Quitting smoking is currently the first and most important part for a COPD treatment plan^40^. Although limited, the PATH dataset does provide some information about pharmacological management of COPD. Based on the fact that many cigarette smokers used ENDS as a cessation tool^41^ and on our results that tobacco flavored ENDS is associated with COPD, longitudinal studies are needed to focus on COPD incidence and explore whether the switching from smoking to vaping has positive or negative effects on COPD, per se, as well as on the effectiveness of COPD treatments.

## CONCLUSIONS

Our study demonstrated that use of tobacco flavored ENDS is significantly associated with self-reported COPD. Among current established ENDS users, regular use of tobacco flavor increased the odds of self-reported COPD regardless of smoking status. Future studies are necessary to confirm the biological and epidemiological association of flavored ENDS use with COPD.
