# Supplementary material for: Association of flavored electronic nicotine delivery system (ENDS) use with self-reported chronic obstructive pulmonary disease (COPD): Results from the Population Assessment of Tobacco and Health (PATH) study, Wave 4
Source: Tob Induc Dis. 2020 Oct 1;18:82. doi: 10.18332/tid/127238 (PMC7549379; doi:10.18332/tid/127238)
Supplement: Supplementary file 1 [file TID-18-82-s1.pdf]

Supplementary Figure S1 & Tables S1-S7

COPD definition:

Figure S1

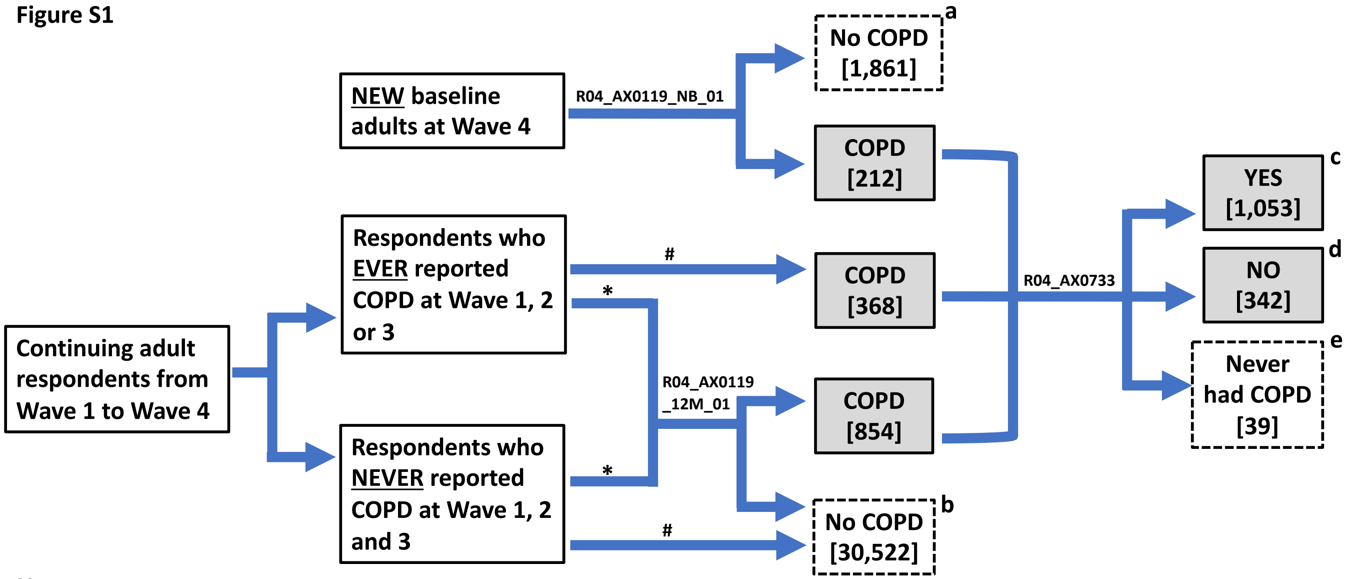

Notes:

1. COPD = Chronic Obstructive Pulmonary Disease;
2. Self-reported COPD prevalent cases : non-COPD cases = (c + d) : (a + b + e);
3. * Respondents who have seen a medical doctor, nurse or other health professional in the past 12 months;
4. # Respondents who NEVER see a medical doctor, nurse or other health professional in the past 12 months.

Figure S1: Flowchart of the process for classifying COPD cases

Figure S1 notes (cont'd):
R04_AX0119_12M_01
In past 12 months, doctor, nurse or other health professional said you had: COPD?

ASK: Continuing adult respondents who have seen a medical doctor, nurse or other health professional in the past 12 months.

Values: 1 = Yes; 2 = No; Missing data.

R03_AX0111_NB_01
Doctor, nurse or other health professional said you had: COPD?

ASK: New baseline adult respondents.

Values: 1 = Yes; 2 = No; Missing data.

R04_AX0733:
Troubled by shortness of breath when hurrying on level ground or walking up a slight hill due to COPD.

ASK: Adult respondents who reported being diagnosed with COPD as of their last completed interview, or new baseline adult respondents who have ever been told they had COPD, or continuing adult respondents who have been told they had COPD in the past 12 months.

Values: 1 = Yes; 2 = No; 3 = I have never had COPD; Missing data.

Table S1. Pearson correlation coefficients matrix of five flavor categories

	Tobacco	Menthol/ mint	Fruit	Candy/ desserts/ sweets	Others
**Tobacco**	1.00				
**Menthol/ mint**	0.11	1.00			
**Fruit**	0.34	0.21	1.00		
**Candy/ desserts/ sweets**	0.21	0.16	0.02	1.00	
**Others**	0.08	0.04	0.03	-0.11	1.00

Table S2. Characteristics of PATH wave 4 adult ever established ENDS users across flavors (weighted % with 95%CI)

	ENDS flavors use				
Variables	Tobacco (n = 588)	Menthol or Mint (n = 672)	Fruit (n = 1,568)	Candy, desserts, or Sweets (n = 1,047)	Others (n = 464)
COPD					
Yes	19.9 (17.2, 23.1)	8.9 (7.3, 10.8)	5.3 (4.3, 6.5)	5.1 (4.2, 6.3)	10.3 (7.0, 15.2)
No	80.1 (79.7, 80.5)	91.1 (90.7, 91.5)	94.7 (94.7, 94.7)	94.9 (94.6, 95.2)	89.7 (89.0, 90.4)
Smoking status					
Current smoker	67.1 (65.7, 68.5)	57.8 (56.2, 59.5)	51.8 (50.5, 53.1)	57.3 (56.1, 58.5)	58.4 (56.7, 60.2)
Former smoker	28.2 (25.6, 31.1)	26.0 (23.4, 29.0)	27.8 (25.1, 30.7)	25.5 (23.3, 28.0)	22.3 (19.4, 25.7)
Non-smoker	4.7 (3.1, 7.2)	16.1 (13.9, 18.8)	20.4 (19.3, 21.7)	17.2 (15.3, 19.3)	19.2 (16.8, 22.0)
Age (years)					
18-44	47.2 (45.3, 49.1)	70.4 (69.0, 71.8)	81.5 (81.3, 81.8)	85.8 (84.9, 86.7)	75.2 (74.8, 75.6)
45-64	41.3 (38.6, 44.2)	24.3 (21.4, 27.7)	16.2 (14.3, 18.4)	13.2 (11.5, 15.1)	20.4 (17.3, 24.1)
65+	11.5 (8.8, 15.1)	5.3 (3.2, 8.9)	2.3 (1.5, 3.4)	1.0 (0.5, 2.1)	4.4 (2.8, 6.9)
Sex					
Male	55.6 (53.2, 58.1)	51.5 (48.8, 54.5)	56.6 (55.8, 57.5)	59.79 (58.37, 61.25)	55.8 (52.2, 59.6)

Female	44.4	48.5	43.4	40.2	44.2
	(42.4, 46.5)	(46.4, 50.6)	(41.7, 45.1)	(38.7, 41.8)	(42.9, 45.5)
Race/Ethnicity					
Non-Hispanic white	83.4	62.8	70.3	75.4	71.4
	(82.2, 84.7)	(61.5, 64.2)	(69.0, 71.6)	(74.4, 76.4)	(69.6, 73.3)
Non-Hispanic black	6.0	13.2	8.8	5.3	7.4
	(4.3, 8.5)	(10.6, 16.3)	(7.5, 10.3)	(4.2, 6.6)	(5.4, 10.2)
Hispanic	5.8	16.2	13.5	10.4	11.6
	(4.6, 7.3)	(13.6, 19.3)	(12.0, 15.2)	(9.0, 12.0)	(9.7, 13.9)
Others	4.7	7.9	7.5	9.0	9.6
	(3.7, 6.1)	(5.7, 10.9)	(6.3, 8.9)	(7.1, 11.2)	(7.6, 12.1)
Asthma					
Yes	16.4	15.1	15.2	16.8	22.5
	(13.8, 19.5)	(13.0, 17.6)	(13.9, 16.6)	(15.2, 18.6)	(19.9, 25.1)
No	83.6	84.9	84.8	83.2	77.5
	(83.1, 84.1)	(83.8, 86.0)	(84.3, 85.3)	(82.6, 83.9)	(76.8, 78.3)
Marijuana use in ENDS					
Never	77.4	73.8	67.1	63.7	67.0
	(76.3, 78.6)	(72.4, 75.1)	(66.0, 68.1)	(62.6, 64.9)	(64.2, 69.9)
Rarely	10.2	12.4	14.9	17.2	14.1
	(8.44, 12.23)	(10.7, 14.5)	(13.6, 16.4)	(15.6, 18.8)	(12.1, 16.4)
Sometimes	7.1	8.2	11.2	12.8	11.3
	(5.6, 9.1)	(6.4, 10.5)	(9.6, 13.0)	(10.8, 15.2)	(8.9, 14.2)
Most of the time	3.0	3.1	4.0	4.4	4.6
	(2.0, 4.4)	(2.1, 4.5)	(3.3, 4.9)	(2.9, 6.6)	(3.4, 6.4)
Every time	2.4	2.6	2.9	2.0	3.1
	(1.5, 3.7)	(1.5, 4.2)	(2.2, 3.8)	(1.4, 2.9)	(1.8, 5.3)
Income					
Less than $10 000	19.2	22.6	14.8	13.7	20.6

	(16.3, 22.6)	(19.7, 26.1)	(13.4, 16.4)	(12.0, 15.6)	(17.1, 24.9)
$10 000 to $24 999	23.3	22.0	24.1	26.1	24.3
	(21.4, 25.5)	(19.8, 24.4)	(22.5, 25.7)	(24.8, 27.5)	(22.3, 26.5)
$25 000 to $49 999	25.3	24.8	24.3	24.1	24.7
	(22.8, 28.1)	(21.8, 28.2)	(22.6, 26.1)	(22.1, 26.4)	(22.7, 26.9)
$50 000 to $99 999	21.2	18.4	22.9	23.8	17.6
	(18.8, 23.8)	(16.5, 20.6)	(21.1, 24.9)	(21.6, 26.3)	(14.8, 20.8)
$100 000 or more	11.1	12.1	13.9	12.3	12.8
	(8.9, 13.9)	(9.8, 15.0)	(12.4, 15.6)	(10.4, 14.6)	(10.7, 15.3)

COPD, Chronic Obstructive Pulmonary Disease; PATH, Population Assessment of Tobacco and Health study.

Table S3. Characteristics of PATH wave 4 adult ever established ENDS users across COPD stratified by smoking status (weighted % with 95%CI)

	Non-smoker (n = 842)			Former smoker (n = 1215)			Current smoker (n = 2772)		
Variables	COPD (n = 23)	Non-COPD (n = 819)	P value	COPD (n = 92)	Non-COPD (n = 1,123)	P value	COPD (n = 300)	Non-COPD (n = 2,472)	P value
Tobacco flavor use			0.31			0.01			<.0001
Yes	29.8 (18.8, 47.5)	5.7 (3.4, 9.5)		43.8 (36.9, 52.1)	18.8 (16.4, 21.6)		47.3 (42.1, 53.2)	21.8 (20.0, 23.2)	
No	70.2 (61.6, 80.0)	94.3 (92.7, 95.9)		56.2 (50.4, 62.7)	81.2 (79.5, 83.1)		52.7 (51.1, 54.3)	78.2 (76.8, 79.7)	
Menthol or mint flavor use			0.60			0.69			0.69
Yes	17.0 (12.6, 23.2)	23.1 (20.0, 26.9)		22.6 (16.2, 31.7)	19.8 (16.8, 23.4)		21.3 (18.4, 24.5)	22.3 (20.6, 24.1)	
No	83.0 (72.4, 95.0)	77.0 (74.7, 79.9)		77.4 (73.2, 81.8)	80.2 (78.3, 82.2)		78.8 (76.2, 81.4)	77.7 (76.9, 78.7)	
Fruit flavor use			0.68			0.01			0.01
Yes	67.1 (55.5, 81.0)	59.6 (56.8, 63.0)		23.6 (18.0, 31.0)	46.1 (41.7, 50.9)		24.6 (21.8, 27.7)	43.1 (40.7, 45.7)	
No	32.9 (22.4, 48.5)	40.4 (37.2, 44.4)		76.4 (67.9, 85.6)	53.9 (49.7, 58.2)		75.4 (72.6, 78.4)	56.9 (54.9, 59.0)	
Candy, desserts or sweets flavor use			0.76			0.03			0.03
Yes	40.3 (22.4, 72.1)	34.3 (31.0, 38.3)		15.8 (11.0, 22.6)	28.9 (25.4, 32.9)		15.1 (12.9, 18.9)	33.0 (30.7, 35.4)	
No	59.7 (58.8, 61.0)	65.7 (62.7, 69.4)		84.2 (81.6, 87.0)	71.1 (67.9, 74.3)		84.4 (82.3, 86.5)	67.0 (65.3, 68.8)	

Other flavors use			0.17			0.61			0.61
Yes	7.3	17.4		8.9	10.9		18.8	13.4	
	(3.4, 16.0)	(14.2, 21.4)		(4.6, 17.6)	(8.8, 13.6)		(12.8, 27.4)	(12.0, 15.0)	
No	92.7	82.6		91.1	89.1		81.3	86.6	
	(89.2, 96.2)	(80.3, 85.4)		(89.8, 92.5)	(87.4, 90.6)		(82.0, 80.6)	(86.1, 87.1)	
Age (years)			0.08			<.0001			<.0001
18-44	35.4	94.0		13.1	68.9		22.1	75.0	
	(33.6, 37.4)	(92.5, 95.4)		(8.0, 21.3)	(66.4, 71.3)		(19.4, 25.1)	(73.6, 76.4)	
45-64	64.6	5.4		48.2	25.2		63.9	22.4	
	(50.7, 82.1)	(3.29, 8.74)		(46.0, 50.2)	(22.6, 28.0)		(61.0, 66.8)	(20.3, 24.6)	
65+	n/a	0.6		38.8	6.0		14.1	2.6	
		(0.23, 1.69)		(31.9, 46.9)	(4.2, 8.4)		(11.4, 17.4)	(1.9, 3.6)	
Sex			0.07			0.01			0.01
Male	29.9	62.8		43.3	59.2		41.6	56.8	
	(23.2, 38.6)	(60.1, 65.6)		(37.8, 49.6)	(56.3, 62.2)		(37.4, 46.3)	(55.6, 58.1)	
Female	70.2	37.2		56.7	40.8		58.4	43.2	
	(58.3, 84.1)	(34.2, 40.7)		(53.9, 59.8)	(37.8, 44.0)		(55.9, 61.0)	(41.7, 44.7)	
Race/Ethnicity			0.60			<.0001			<.0001
Non-Hispanic white	55.8	51.1		89.6	75.4		81.9	75.5	
	(40.2, 77.1)	(48.3, 54.5)		(88.5, 90.8)	(73.6, 77.3)		(80.3, 83.4)	(74.1, 76.9)	
Non-Hispanic black	18.6	15.1		5.1	6.9		8.7	7.4	
	(13.7, 25.4)	(12.0, 18.9)		(2.5, 10.5)	(5.4, 8.8)		(6.7, 11.5)	(6.4, 8.6)	
Hispanic	13.8	27.2		3.2	10.1		3.6	10.2	
	(7.5, 25.3)	(23.1, 32.0)		(1.5, 6.9)	(8.7, 11.8)		(2.3, 5.7)	(9.1, 11.5)	
Others	11.9	6.6		2.1	7.6		5.8	6.9	

	(7.4, 19.1)	(4.8, 9.2)		(0.8, 5.7)	(5.9, 9.6)		(4.1, 8.1)	(5.8, 8.1)	
Asthma			0.08			0.002			0.002
Yes	55.9	20.0		34.0	11.7		38.7	12.0	
	(43.6, 71.4)	(17.2, 23.2)		(29.1, 39.7)	(10.2, 13.6)		(35.2, 42.5)	(10.8, 13.4)	
No	44.2	80.1		66.0	88.3		61.3	88.0	
	(39.8, 49.2)	(78.1, 82.4)		(60.1, 72.4)	(87.8, 88.9)		(59.0, 63.7)	(87.1, 88.8)	
Marijuana use in ENDS			0.57			0.52			0.52
Never	53.7	60.3		85.4	78.3		83.6	73.5	
	(49.5, 58.4)	(57.4, 63.6)		(82.5, 88.4)	(76.7, 80.0)		(81.7, 85.6)	(71.9, 75.0)	
Rarely	2.8	15.1		6.2	10.1		9.8	12.3	
	(0.7, 10.8)	(12.5, 18.2)		(3.3, 11.8)	(8.5, 12.0)		(7.8, 12.4)	(11.0, 13.7)	
Sometimes	22.6	11.6		3.0	6.1		3.2	8.2	
	(8.2, 61.7)	(9.2, 14.6)		(1.2, 7.5)	(4.4, 8.3)		(2.0, 5.2)	(7.0, 9.6)	
Most of the time	9.3	6.8		3.1	3.3		1.5	3.0	
	(5.2, 16.8)	(4.8, 9.5)		(1.2, 7.8)	(2.1, 5.2)		(0.7, 3.3)	(2.4, 3.7)	
Every time	11.7	6.3		2.3	2.3		1.8	3.1	
	(7.3, 18.9)	(4.7, 8.5)		(0.6, 8.7)	(1.6, 3.4)		(0.9, 3.6)	(2.3, 4.0)	
Income						0.03			0.03
Less than $10 000	65.9	20.1		8.6	9.3		28.1	16.6	
	(55.0, 78.8)	(16.7, 24.1)		(5.6, 13.1)	(7.5, 11.5)		(23.2, 34.2)	(15.3, 18.0)	
$10 000 to $24 999	30.3	23.8		35.7	19.1		33.9	24.0	
	(21.0, 43.9)	(20.6, 27.5)		(30.9, 42.3)	(16.4, 22.2)		(29.9, 38.3)	(22.4, 25.6)	
$25 000 to $49 999	2.2	22.2		20.9	26.1		20.8	26.1	
	(0.5, 10.5)	(19.0, 26.1)		(15.3, 28.7)	(23.5, 29.5)		(18.4, 23.7)	(24.0, 28.4)	
$50 000 to $99 999	1.5	18.0		16.1	27.5		13.2	23.0	

	(0.2, 9.7)	(15.3, 21.3)	(11.0, 23.5)	(25.1, 30.3)	(10.2, 17.0)	(21.0, 25.1)
$100 000 or more	n/a	16.0	18.8	17.8	4.0	10.4
		(13.4, 19.1)	(14.4, 24.5)	(15.7, 20.2)	(2.3, 7.0)	(8.9, 12.0)

COPD, Chronic Obstructive Pulmonary Disease; PATH, Population Assessment of Tobacco and Health study.

n/a: number of COPD cases is insufficient for calculation.

Table S4. Characteristics of PATH wave 4 adult participants across COPD stratified by ENDS use status (weighted % with 95%CI)

	All established ENDS users (n = 4,909)			Current established ENDS users (n = 1,988)			Former established ENDS users (n = 2,921)		
Variables	COPD (n = 418)	Non-COPD (n = 4,491)	P value	COPD (n = 152)	Non-COPD (n = 1,836)	P value	COPD (n = 266)	Non-COPD (n = 2,655)	P value
Tobacco flavor use									
Yes	45.18 (41.20, 49.74)	18.09 (16.89, 19.39)	<.0001	43.40 (37.03, 50.80)	16.02 (14.44, 17.79)	<.0001	48.48 (43.24, 54.23)	21.47 (19.23, 23.96)	<.0001
No	54.72 (52.66, 56.89)	81.91 (80.90, 82.93)		56.6 (55.60, 57.68)	83.98 (82.84, 85.13)		51.52 (46.18, 57.44)	78.53 (76.65, 80.38)	
Menthol or mint flavor use			0.89			0.66			0.47
Yes	21.26 (18.87, 23.96)	21.64 (20.43, 22.94)		20.79 (18.06, 23.97)	19.32 (17.49, 21.35)		22.05 (16.95, 28.66)	25.45 (22.93, 28.22)	
No	78.74 (77.14, 80.37)	78.36 (77.59, 79.18)		79.21 (78.10, 80.36)	80.68 (79.46, 81.95)		77.95 (74.97, 81.20)	74.55 (72.50, 76.58)	

Fruit flavor use			<.0001			0.0009			0.0009
Yes	26.39 (23.58, 29.55)	46.85 (45.07, 48.70)		27.43 (23.18, 32.46)	49.34 (47.59, 51.20)		24.61 (19.58, 30.93)	42.77 (39.35, 46.35)	
No	73.61 (69.22, 78.20)	53.15 (51.67, 54.69)		72.57 (65.55, 80.16)	50.66 (48.98, 52.45)		75.39 (71.36, 79.58)	57.23 (54.24, 60.26)	
Candy, desserts or sweets flavor use			<.0001			0.0025			0.0002
Yes	17.39 (15.15, 19.97)	32.04 (30.16, 34.04)		19.88 (16.64, 23.78)	33.65 (31.64, 35.80)		13.13 (8.39, 20.54)	29.42 (26.55, 32.54)	
No	82.61 (80.96, 84.30)	67.96 (66.40, 69.53)		80.12 (76.19, 84.18)	66.35 (64.73, 68.05)		86.87 (85.38, 88.38)	70.58 (68.01, 73.12)	
Other flavors use			0.52			0.22			0.02
Yes	15.57 (10.87, 22.26)	13.45 (12.27, 14.74)		19.17 (12.24, 29.91)	12.10 (10.57, 13.86)		9.43 (5.91, 15.04)	15.66 (13.57, 18.06)	
No	84.43 (86.14, 82.81)	86.55 (85.90, 87.23)		80.83 (82.40, 79.38)	87.90 (87.28, 88.58)		90.57 (90.71, 90.47)	84.34 (82.71, 85.90)	
Smoking status			<.0001			0.0048			<.0001

Current smoker	70.21	57.46		64.04	53.09		73.93	60.49	
	(69.80, 70.65)	(56.26, 58.70)		(60.02, 68.29)	(51.06, 55.21)		(73.55, 74.35)	(58.63, 62.36)	
Former smoker	25.28	28.35		29.86	31.4		22.51	26.24	
	(22.35, 28.58)	(26.98, 29.79)		(25.99, 34.33)	(29.30, 33.67)		(18.81, 26.92)	(24.37, 28.23)	
Non-smoker	4.52	14.19		6.1	15.51		3.56	13.27	
	(2.85, 7.15)	(13.13, 15.33)		(2.89, 12.86)	(13.79, 17.45)		(2.22, 5.71)	(11.96, 14.73)	
Age (years)			<.0001			<.0001			<.0001
18-44	20.71	76.12		19.03	75.85		21.72	76.30	
	(18.04, 23.77)	(75.00, 77.23)		(16.43, 22.07)	(74.01, 77.48)		(17.93, 26.31)	(75.00, 77.60)	
45-64	59.73	20.64		58.50	20.61		60.47	20.66	
	(58.15, 61.35)	(19.21, 22.18)		(56.95, 60.14)	(18.65, 22.78)		(58.10, 62.94)	(18.87, 22.62)	
65+	19.57	3.24		22.48	3.54		17.80	3.03	
	(16.29, 23.50)	(2.60, 4.03)		(17.10, 29.51)	(2.42, 5.17)		(14.67, 21.60)	(2.36, 3.91)	
Sex			<.0001			0.02			0.02
Male	41.73	58.34		36.05	61.29		45.17	56.32	
	(37.51, 46.39)	(57.45, 59.28)		(27.77, 46.65)	(59.65, 63.01)		(40.58, 50.24)	(54.86, 57.82)	
Female	58.27	41.66		63.95	38.71		54.83	43.68	

	(56.44, 60.17)	(40.55, 42.81)		(63.61, 64.36)	(37.03, 40.52)		(50.62, 59.34)	(42.10, 45.33)	
Race/Ethnicity			<.0001			0.0032			<.0001
Non-Hispanic white	82.70	71.72		82.79	74.78		82.65	69.60	
	(81.56, 83.86)	(70.52, 72.92)		(79.99, 85.64)	(72.82, 76.72)		(81.02, 84.29)	(68.07, 71.14)	
Non-Hispanic black	8.27	8.49		7.75	7.35		8.60	9.28	
	(6.69, 10.24)	(7.53, 9.57)		(5.54, 10.85)	(5.92, 9.10)		(6.42, 11.53)	(8.25, 10.45)	
Hispanic	3.94	12.74		3.94	10.84		3.94	14.04	
	(2.80, 5.53)	(11.64, 13.94)		(2.30, 6.75)	(9.43, 12.48)		(2.52, 6.14)	(12.56, 15.69)	
Others	5.09	7.05		5.52	7.03		4.82	7.07	
	(3.82, 6.78)	(6.28, 7.92)		(3.60, 8.49)	(5.78, 8.56)		(3.26, 7.12)	(6.10, 8.19)	
Asthma			<.0001			<.0001			<.0001
Yes	38.49	13.07		37.22	11.85		39.27	13.91	
	(36.43, 40.68)	(12.16, 14.05)		(31.78, 43.55)	(10.56, 13.31)		(36.75, 41.96)	(12.73, 15.20)	
No	61.51	86.93		62.78	88.15		60.73	86.09	
	(59.13, 63.97)	(86.44, 87.44)		(62.02, 63.62)	(87.58, 88.80)		(57.11, 64.55)	(85.23, 86.95)	

Marijuana use in ENDS			<.0001			0.02			0.0024
Never	82.81 (81.26, 84.38)	73.00 (71.87, 74.13)		82.16 (79.89, 84.49)	71.01 (69.47, 72.61)		83.21 (81.33, 85.10)	74.37 (72.96, 75.76)	
Rarely	8.55 (6.89, 10.62)	11.97 (11.06, 12.97)		7.97 (5.40, 11.77)	14.19 (12.59, 16.01)		8.91 (6.60, 12.04)	10.44 (9.36, 11.65)	
Sometimes	4.02 (2.61, 6.22)	8.07 (7.16, 9.11)		5.98 (3.08, 11.61)	9.47 (8.12, 11.06)		2.83 (1.53, 5.24)	7.11 (6.20, 8.16)	
Most of the time	2.24 (1.18, 4.26)	3.61 (3.04, 4.30)		2.15 (0.99, 4.66)	2.91 (2.21, 3.85)		2.29 (0.92, 5.67)	4.10 (3.32, 5.05)	
Every time	2.37 (1.49, 3.78)	3.34 (2.85, 3.92)		1.73 (0.72, 4.15)	2.41 (1.70, 3.41)		2.76 (1.60, 4.78)	3.99 (3.26, 4.88)	
Income			<.0001			0.0065			<.0001
Less than $10 000	24.95 (21.78, 28.58)	14.97 (13.83, 16.19)		21.86 (16.67, 28.63)	13.52 (11.81, 15.49)		26.80 (22.89, 31.35)	15.95 (14.50, 17.54)	

$10 000 to $24 999	33.97	22.54	33.25	22.19	34.40	22.79
	(30.57, 37.73)	(21.20, 23.97)	(29.69, 37.26)	(20.33, 24.24)	(29.58, 39.96)	(20.92, 24.80)
$25 000 to $49 999	20.03	25.73	18.27	25.40	20.81	25.96
	(17.87, 22.46)	(24.37, 27.17)	(14.48, 24.22)	(23.41, 27.57)	(18.19, 23.82)	(24.15, 27.88)
$50 000 to $99 999	13.62	23.45	15.99	24.29	12.20	22.87
	(10.94, 16.95)	(21.79, 25.12)	(12.65, 20.23)	(22.36, 26.39)	(9.18, 16.22)	(20.93, 24.98)
$100 000 or more	7.43	13.31	10.18	14.60	5.79	12.43
	(5.27, 10.48)	(12.11, 14.63)	(6.70, 15.47)	(12.92, 16.50)	(3.88, 8.65)	(11.07, 13.97)

COPD, Chronic Obstructive Pulmonary Disease; PATH, Population Assessment of Tobacco and Health study.

Table S5. (Model 1) Adjusted ORs (with 95% CI)* of COPD prevalence associated with ENDS flavors stratified by vaping and smoking status

	Former established ENDS users			Current established ENDS users		
	Non-smoker	Former-smoker	Current-smoker	Non-smoker	Former-smoker	Current-smoker
Tobacco	n/a	2.45 (0.26, 24.22)	2.06 (0.96, 4.44)	n/a	2.77 (0.90, 8.54)	**2.78 (1.46, 5.30)**
Menthol or mint	n/a	0.60 (0.06, 6.25)	1.00 (0.39, 2.62)	0.27 (0.00, 16.43)	1.76 (0.86, 5.55)	1.07 (0.60, 1.93)
Fruit	n/a	0.97 (0.09, 10.99)	0.65 (0.27, 1.58)	3.59 (0.28, 46.82)	0.46 (0.12, 1.80)	0.63 (0.38, 1.06)
Candy, desserts, or sweets	n/a	2.76 (0.64, 10.41)	0.25 (0.08, 0.76)	n/a	0.23 (0.03, 2.11)	0.63 (0.38, 1.04)
Others	n/a	0.18 (0.01, 3.26)	1.27 (0.49, 3.34)	n/a	1.86 (0.48, 7.28)	**2.23 (1.06, 5.01)**

* Users of a specific flavor vs non-users of this specific flavor as reference. The adjusted ORs controlled the effect of all flavors other than the interested specific flavor.
n/a: number of COPD prevalent case is insufficient for calculation.

Table S6. (Model 4) Adjusted ORs (with 95% CI)of COPD prevalence associated with ENDS flavors stratified by vaping status and asthma

	Users of a specific flavor vs non-users of this specific flavor as reference				
	Tobacco	Menthol or mint	Fruit	Candy, desserts or sweets	Others
**With history of asthma**					
Ever established ENDS users	1.91 (0.71, 5.15)	0.98 (0.37, 2.60)	0.99 (0.43, 2.27)	0.80 (0.34, 1.88)	1.14 (0.52, 2.50)
Former established ENDS users	n/a	n/a	n/a	n/a	n/a
Current established ENDS users	2.83 (0.69, 11.50)	0.85 (0.24, 2.95)	0.77 (0.26, 2.24)	1.16 (0.36, 3.69)	0.97 (0.35, 2.68)
**Without history of asthma**					
Ever established ENDS users	1.38 (0.73, 2.61)	1.00 (0.50, 2.01)	0.78 (0.38, 1.61)	0.72 (0.44, 1.20)	1.07 (0.62, 1.84)
Former established ENDS users	0.63 (0.14, 2.87)	0.34 (0.05, 2.42)	0.48 (0.08, 3.02)	0.40 (0.06, 2.54)	0.26 (0.03, 2.14)
Current established ENDS users	1.83 (0.88, 3.81)	1.44 (0.64, 3.24)	0.88 (0.46, 1.67)	0.86 (0.39, 1.91)	2.01 (0.75, 5.43)

The adjusted ORs controlled the effects of all other flavors, smoking status, sex, age, race/ethnicity, and income level.
n/a: number of COPD prevalent case is insufficient for calculation.

Table S7. (Model 5) Adjusted ORs (with 95% CI)of COPD prevalence associated with ENDS flavors stratified by vaping status and sex

	Users of a specific flavor vs non-users of this specific flavor as reference				
	Tobacco	Menthol or mint	Fruit	Candy, desserts or sweets	Others
**Male**					
Ever established ENDS users	1.78 (0.84, 3.77)	0.73 (0.25, 2.13)	0.51 (0.19, 1.38)	0.58 (0.24, 1.39)	2.22 (1.09, 4.49)
Former established ENDS users	1.86 (0.27, 12.89)	0.43 (0.04, 5.17)	1.81 (0.22, 14.77)	0.35 (0.05, 2.66)	1.62 (0.20, 12.94)
Current established ENDS users	n/a	n/a	n/a	n/a	n/a
**Female**					
Ever established ENDS users	1.51 (0.78, 2.89)	1.27 (0.72, 2.24)	1.03 (0.54, 1.98)	1.02 (0.61, 1.71)	0.58 (0.27, 1.25)
Former established ENDS users	0.86 (0.20, 3.67)	0.90 (0.19, 4.18)	0.70 (0.15, 3.19)	0.78 (0.13, 4.78)	0.32 (0.06, 1.66)
Current established ENDS users	2.37 (0.99, 5.68)	1.63 (0.79, 3.38)	1.31 (0.60, 2.86)	1.37 (0.76, 2.48)	0.87 (0.29, 2.63)

The adjusted ORs controlled the effects of all other flavors, smoking status, sex, age, race/ethnicity, and income level.
n/a: number of COPD prevalent case is insufficient for calculation.

© 2020 Shi H. et al.
